# Hepatitis C-Induced Immunoglobulin A (IgA) Nephropathy: An Uncommon Cause of Hematuria

**DOI:** 10.7759/cureus.70168

**Published:** 2024-09-25

**Authors:** Jagadeswar Kakumani, Krishna Geetha Narne, Amukthamalyada Koduri, Prem Balaji Reddy Lankapothu, Magesh Kumar S

**Affiliations:** 1 Internal Medicine, Saveetha Medical College and Hospital, Saveetha Institute of Medical and Technical Sciences, Saveetha University, Chennai, IND; 2 General Medicine, Saveetha Medical College and Hospital, Saveetha Institute of Medical and Technical Sciences, Saveetha University, Chennai, IND

**Keywords:** hematuria, hepatitis c (hcv) infection, iga nephropathy (igan), immunoglobulin a., membranoproliferative glomerulonephritis (mpgn), proteinuria

## Abstract

Immunoglobulin (Ig)A nephropathy, also known as Berger’s disease, is characterized by IgA deposits in the kidney's mesangium and can lead to serious outcomes, including rapidly progressive glomerulonephritis. While Hepatitis C virus (HCV) infection is commonly associated with liver involvement, it is also linked to various renal pathologies, including membranous nephropathy and membranoproliferative glomerulonephritis. However, IgA nephropathy secondary to HCV is rare. This case study involves a 24-year-old male with a known history of untreated Hepatitis C, who presented with hematuria and foamy urine. Laboratory findings revealed increased levels of serum creatinine and urea, significant proteinuria, and a high HCV RNA viral load. A renal biopsy confirmed the diagnosis of IgA nephropathy with strong IgA and C3 deposits observed on immunofluorescence. Treatment with angiotensin receptor blockers and antiviral therapy led to substantial symptomatic improvement and stabilization of renal function. This case underscores the critical importance of considering IgA nephropathy in the differential diagnosis of renal complications in HCV-infected patients. Early detection and treatment of Hepatitis C are vital, as timely intervention can markedly enhance patient outcomes and mitigate the risk of serious complications. A heightened awareness of the potential renal implications of HCV emphasizes the urgency of proactive monitoring and swift management to safeguard kidney function and overall health.

## Introduction

Berger’s disease, also known as immunoglobulin (Ig)A nephropathy, is a major and complex kidney disorder that occurs when IgA is deposited in the mesangium, a central part of the glomeruli, which are tiny filtering units in the kidneys [1]. It is one of the most common types of primary glomerulonephritis worldwide, with a prevalence of about two to three cases per 100,000 people in Western countries and up to 10-20 cases per 100,000 in East Asia. Rates can vary due to genetic and environmental factors [2]. The condition involves the accumulation of IgA immune complexes within the glomeruli, leading to mesangial cell proliferation and glomerular inflammation [3]. The clinical presentations of IgA nephropathy vary widely, making early diagnosis challenging. In its mildest form, it may present as asymptomatic microscopic hematuria, where blood is present in the urine but only visible under a microscope [4]. However, in more severe cases, patients may experience gross hematuria, which is visible without assistance and often coincides with upper respiratory infections. If left untreated, it can progress to rapidly progressive glomerulonephritis, a type of kidney inflammation that can cause acute kidney injury and eventual renal failure. A significant proportion of individuals with IgA nephropathy may progress to chronic kidney disease (CKD) or end-stage renal disease (ESRD), necessitating dialysis or kidney transplantation [5]. This progression is particularly concerning in the context of Hepatitis C virus (HCV) infection, which primarily affects the liver but has been associated with renal complications.

Hepatitis C virus (HCV) is a single-stranded RNA virus that primarily affects the liver [6]. HCV has become a significant global health issue, impacting millions worldwide, especially those with limited access to medical care. Although HCV is well-known for causing chronic liver diseases such as cirrhosis and hepatocellular carcinoma, it also has many extrahepatic manifestations, including renal complications [7]. Various renal diseases associated with chronic HCV infection can significantly affect a patient’s prognosis. The most frequently reported renal pathology associated with HCV is membranoproliferative glomerulonephritis (MPGN), often accompanied by mixed cryoglobulinemia, where cryoglobulins precipitate in the cold, forming abnormal proteins that can block blood vessels and lead to vasculitis [8]. MPGN manifests as thickened capillary walls and mesangial expansion in the glomeruli due to immune complex deposition. Another kidney complication associated with HCV is membranous glomerulopathy, characterized by thickening of the glomerular basement membrane with minimal glomerular cell proliferation. Both conditions result in significant proteinuria, hematuria, and progressive renal insufficiency [9]. Although there is well-documented evidence linking HCV to these renal pathologies, the occurrence of IgA nephropathy secondary to HCV infection is extremely rare. Most instances of HCV-related renal disease are mediated via immune complexes, although the mechanisms by which HCV may influence IgA nephropathy development remain speculative [10]. It is thought that prolonged HCV infection may alter the immune response, promoting the formation of abnormal IgA immune complexes that deposit in the kidneys, triggering the pathological processes characteristic of IgA nephropathy. Recognizing IgA nephropathy in patients with HCV is vital, as early diagnosis and intervention can significantly improve outcomes. Clinicians should maintain a high index of suspicion for IgA nephropathy in HCV-infected individuals to ensure timely management and reduce the risk of severe kidney damage.

## Case presentation

This is the case of a 24-year-old male patient with Hepatitis C who presented to our clinic with complaints of gradually occurring hematuria and foamy urine for approximately one month. However, the patient had not received any antiviral therapy despite his Hepatitis C status prior to this visit. The pale appearance of the patient during general examination indicated anemia, while bilateral pedal edema suggested fluid retention. On systemic examination, however, no other significant findings were seen. Upon presentation, vital signs showed blood pressure at 160/90 mmHg, pulse rate at 70 beats per minute, and respiratory rate at 16 breaths per minute, indicating hypertension. Based on these symptoms and clinical findings, laboratory investigations were carried out, revealing several abnormalities. A low level of haemoglobin was observed, confirming that the patient had anemia. The total leukocyte count and platelet count were within the normal range. However, renal function test results indicated increased serum urea levels , as well as serum creatinine levels elevated , indicating kidney damage. Liver function tests revealed mildly raised liver enzymes, and total protein and albumin were reduced, suggesting nephrotic syndrome (Table 1). Additionally, further urine examination showed spot polymerase chain reaction (PCR) for a protein-creatinine ratio of 1:4, with urinary protein excretion exceeding 1g/day according to a 24-hour urine collection. A urine examination showed proteinuria (2+), indicating a moderate level of protein and 20-25 dysmorphic red blood cells (RBCs) per high power field, indicative of glomerular damage typical for glomerulonephritis (Table 1).

**Table 1:** Laboratory values SGOT: Serum glutamic-oxaloacetic transaminase; AST: Aspartate aminotransferase; SGPT: Serum glutamate pyruvate transaminase; ALT: Alanine aminotransferase.

Test	Result	Normal Range
Hemoglobin	8.2 mg/dL	12.0-16.0 g/dL (female), 13.5-17.5 g/dL (male)
Total Leukocyte Count	10,120 cells/mm³	4,000-11,000 cells/mm³
Platelet Count	399,000/mm³	150,000-450,000/mm³
Serum Urea	61 mg/dL	7-20 mg/dL
Serum Creatinine	4.6 mg/dL	0.6-1.2 mg/dL
Total Bilirubin	1.2 mg/dL	0.1-1.2 mg/dL
Direct Bilirubin	0.6 mg/dL	0.0-0.3 mg/dL
SGOT (AST)	65 U/L	0-40 U/L
SGPT (ALT)	75 U/L	0-40 U/L
Total Protein	5.8 g/dL	6.0-8.0 g/dL
Albumin	2.8 g/dL	3.5-5.0 g/dL
Urine Protein-Creatinine Ratio	1.4	<0.2
24-Hour Urine Protein Excretion	>1 g/day	<0.3 g/day
Urine Protein	2+	Negative
Dysmorphic Red Blood Cells	20-25 per high power field	<5 per high power field

**Figure 1:**
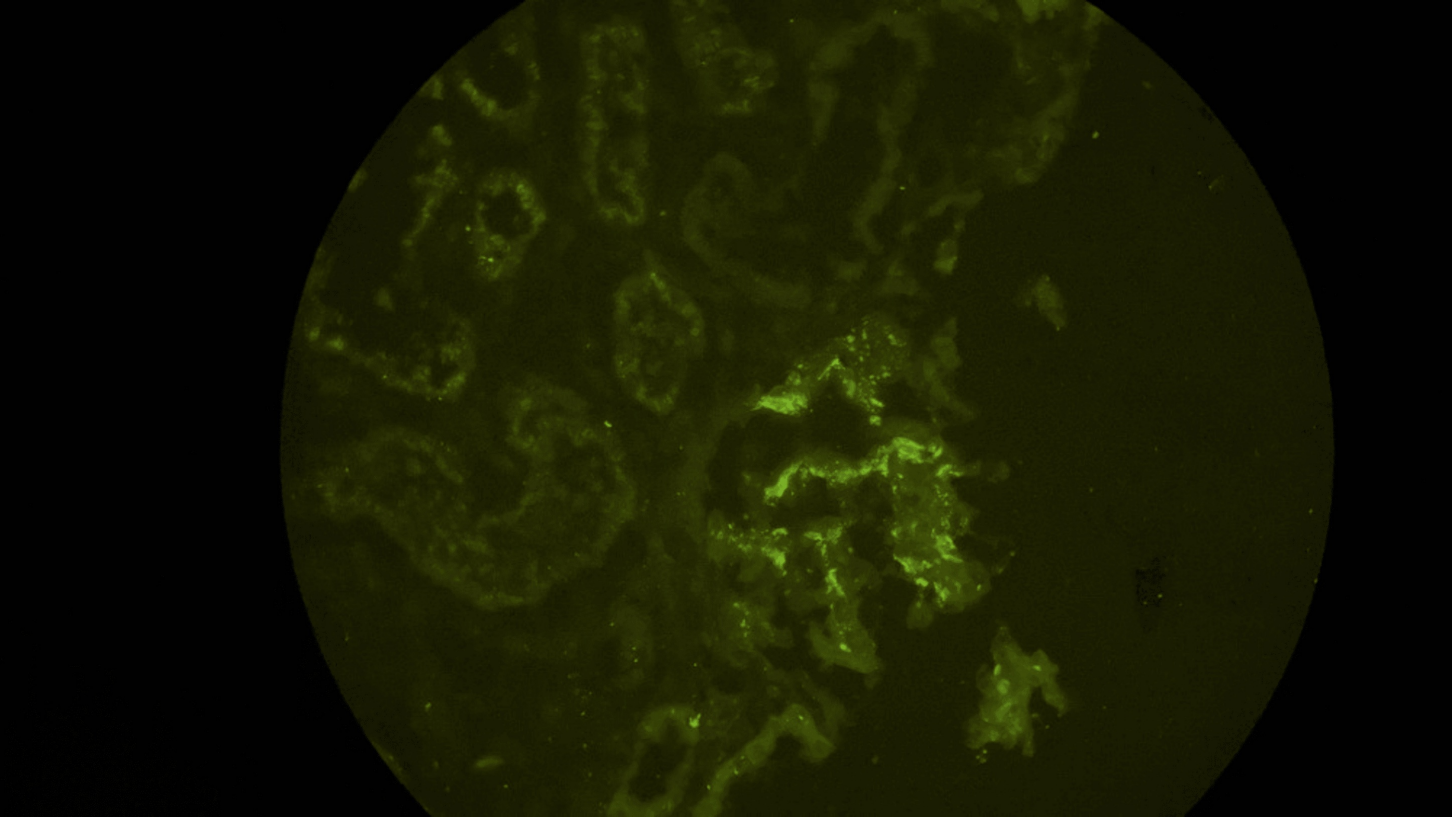
Immunofluorescence image The image shows immunoglobulin (Ig)A deposition in mesangium.

An ultrasound scan of the abdomen revealed normal-sized kidneys with maintained corticomedullary differentiation, noting no structural defects. Because the patient had Hepatitis C, an HCV RNA viral load test was conducted, which showed a very high viral load of 1,456,387 IU/L. However, serum cryoglobulin levels were normal despite the high viral load, thereby excluding cryoglobulinemia, which can sometimes be associated with Hepatitis C [11]. A renal biopsy was performed due to abnormal renal findings in order to ascertain the cause of the patient’s symptoms. Immunofluorescence studies on the tissue showed strong IgA deposits (3+) and moderate C3 deposits (2+) along the glomerular capillary walls and mesangium (Figure 1). The granular positivity observed within these areas indicates immune complex deposition, consistent with IgA nephropathy.

**Figure 2:**
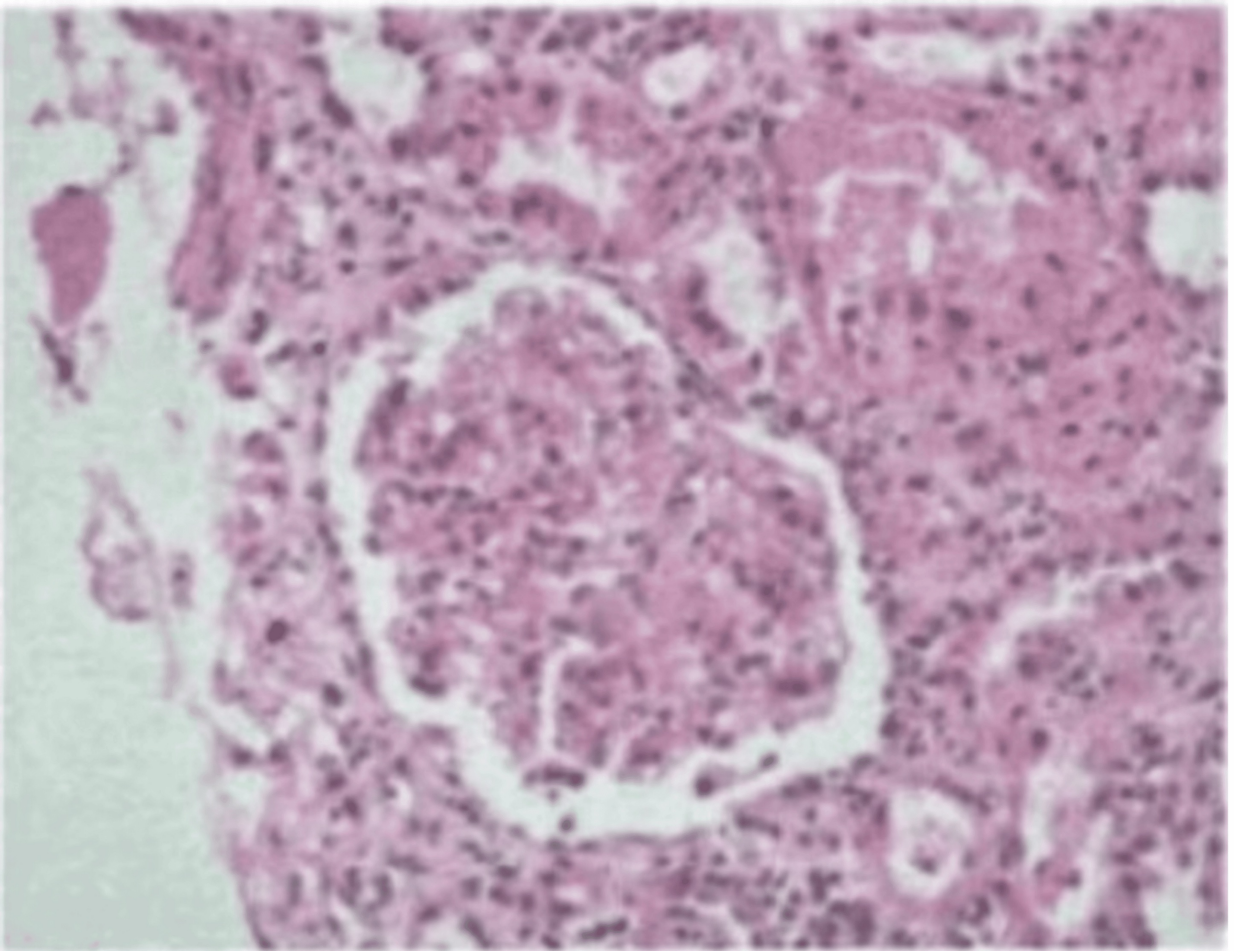
Histopathological examination The histopathological image shows endocapillary hypercellularity.

The biopsy also had a MEST-C score of M1E1S0T0C0 (M: mesangial hypercellularity, E: endocapillary hypercellularity, S: segmental glomerulosclerosis, T: tubular atrophy/interstitial fibrosis, C: cellular crescents score is essential for assessing IgA nephropathy severity, guiding treatment decisions, and standardizing diagnosis), indicating both mesangial hypercellularity and endocapillary hypercellularity (Figure 2) without segmental sclerosis, tubular atrophy, or crescents.

These findings confirmed IgA nephropathy as a complication of chronic Hepatitis C infection. Following his diagnosis, treatment was promptly initiated. Telmisartan 40 mg orally once daily was started, preferred for IgA nephropathy due to its superior proteinuria reduction and renal protection, while alternatives like losartan, valsartan, and irbesartan may require more frequent dosing or provide less renal benefit. Antiviral drugs were also recommended for him, considering his elevated HCV RNA viral level after his diagnosis with Hepatitis C infection. The patient responded well to the treatment regimen. His hematuria resolved, and his subsequent renal function tests improved greatly, with serum urea level dropping to 34 mg/dL and serum creatinine levels reducing to 1.5 mg/dL. As a result of these improvements, the patient was discharged in stable condition and advised to continue regular follow-ups to monitor kidney function and adhere to Hepatitis C treatment.

## Discussion

This case underscores the significant, albeit uncommon, association between chronic Hepatitis C virus (HCV) infection and IgA nephropathy. Recognizing this link is crucial because, while conditions like membranoproliferative glomerulonephritis (MPGN) and membranous glomerulopathy are more frequently associated with HCV, the presence of IgA nephropathy may lead to different management strategies and outcomes. Previous studies have primarily focused on the more common glomerular diseases linked to HCV, such as MPGN, where cryoglobulinemia often plays a pivotal role [11]. However, IgA nephropathy in the context of HCV remains underreported, with only a limited number of case reports and small case series available in the literature.

A comparative analysis of these studies reveals a consistent observation: HCV-infected patients presenting with IgA nephropathy often demonstrate mesangial IgA deposition without clear evidence of the cryoglobulinemic involvement typically seen in MPGN. Several studies have explored the association between Hepatitis C virus (HCV) infection and various renal pathologies, including IgA nephropathy. Fabrizi et al. (2019) reviewed glomerular diseases linked to HCV, such as IgA nephropathy and membranoproliferative glomerulonephritis (MPGN), highlighting the role of direct-acting antivirals in improving renal outcomes [12]. Ozkok and Yildiz (2024) presented a case of HCV-associated IgA nephropathy with mesangial IgA deposition, aligning with similar reports in the literature [13]. The KDIGO 2018 guidelines and a meta-analysis by Martin et al. (2022) also underscore the importance of considering IgA nephropathy in HCV-infected patients with renal symptoms, despite its lower prevalence compared to MPGN [14].

Tang and Lai (2013) provided early insights into the spectrum of HCV-associated glomerulonephritis, reinforcing the need for clinicians to maintain a broad differential when managing renal manifestations in HCV patients. These studies collectively emphasize the diverse renal presentations in HCV and the critical role of timely diagnosis and management [15]. The pathogenic mechanism of HCV-associated IgA nephropathy is not fully understood, but it is hypothesized that persistent HCV infection may trigger an aberrant immune response, leading to the deposition of IgA-containing immune complexes in the glomeruli [11]. This deposition subsequently initiates an inflammatory response, causing glomerular injury and clinical manifestations of IgA nephropathy. This mechanism contrasts with the pathogenesis of HCV-associated MPGN, where immune complex deposition typically involves IgG and cryoglobulins rather than IgA. Histopathological findings in this case highlight key features of IgA nephropathy, including positive immunofluorescence staining for IgA and C3 in the mesangium and glomerular capillary walls. The MEST-C score further aids in grading the severity of the disease, thus guiding therapeutic decisions. In comparison to similar cases reported in the literature, our findings align with those indicating mesangial IgA deposition without the proliferative changes more characteristic of MPGN or membranous glomerulopathy linked to HCV. The case emphasizes the critical importance of early detection and prompt management of IgA nephropathy secondary to HCV infection. Antiviral therapy, in conjunction with supportive measures such as angiotensin receptor blockers, has been shown to improve renal outcomes and mitigate progression to end-stage renal disease [12]. This aligns with the broader literature on the management of HCV-associated renal disease, where timely intervention has consistently been associated with better clinical outcomes.

## Conclusions

This case report discusses a rare presentation of IgA nephropathy due to chronic HCV infection, highlighting the importance of considering this diagnosis in HCV-infected patients with renal symptoms. Timely and aggressive therapy targeting both the underlying viral disease and kidney complications is essential to prevent chronic kidney failure or end-stage renal disease. Additionally, the case contributes to the literature on the extrahepatic manifestations of Hepatitis C, emphasizing the need for multidisciplinary management of HCV, especially in patients with concurrent renal involvement. Future studies should focus on elucidating the immune response mechanisms and cytokine profiles involved in HCV-associated IgA nephropathy, as well as evaluating the long-term renal benefits of direct-acting antivirals combined with angiotensin receptor blockers. Personalized treatment approaches based on patient-specific factors should also be prioritized to optimize management strategies.
